# Tinnitus, Unipolar Brush Cells, and Cerebellar Glutamatergic Function in an Animal Model

**DOI:** 10.1371/journal.pone.0064726

**Published:** 2013-06-13

**Authors:** Carol A. Bauer, Kurt W. Wisner, Joan S. Baizer, Thomas J. Brozoski

**Affiliations:** 1 Department of Otolaryngology, Southern Illinois University School of Medicine, Springfield, Illinois, United States of America; 2 Department of Physiology and Biophysics, University at Buffalo, Buffalo, New York, United States of America; University of Valencia, Spain

## Abstract

Unipolar brush cells (UBCs) are excitatory interneurons found in the dorsal cochlear nucleus (DCN) and the granule cell layer of cerebellar cortex, being particularly evident in the paraflocculus (PFL) and flocculus (FL). UBCs receive glutamatergic inputs and make glutamatergic synapses with granule cells and other UBCs. It has been hypothesized that UBCs comprise local networks of tunable feed-forward amplifiers. In the DCN they might also participate in feed-back amplification of signals from higher auditory centers. Recently it has been shown that UBCs, in the vestibulocerebellum and DCN of adult rats, express doublecortin (DCX), previously considered a marker of newborn and migrating neurons. In an animal model, both the DCN, and more recently the PFL, have been implicated in contributing to the sensation of acoustic-exposure-induced tinnitus. These studies support the working hypothesis that tinnitus emerges after loss of peripheral sensitivity because inhibitory processes homeostatically down regulate, and excitatory processes up regulate. Here we report the results of two sequential experiments that examine the potential role of DCN and cerebellar UBCs in tinnitus, and the contribution of glutamatergic transmission in the PFL. In Experiment 1 it was shown that adult rats with psychophysical evidence of tinnitus induced by a single unilateral high-level noise exposure, had elevated DCX in the DCN and ventral PFL. In Experiment 2 it was shown that micro-quantities of glutamatergic antagonists, delivered directly to the PFL, reversibly reduced chronically established tinnitus, while similarly applied glutamatergic agonists induced tinnitus-like behavior in non-tinnitus controls. These results are consistent with the hypothesis that UBC up regulation and enhanced glutamatergic transmission in the cerebellum contribute to the pathophysiology of tinnitus.

## Introduction

Tinnitus, or ringing in the ears, is experienced by a significant portion of the general adult population. The prevalence of tinnitus derived from population-based estimates varies with the age of subjects and the definition of tinnitus. Nondahl estimated that 8.2% of adults experience moderately severe tinnitus with a 5-year incidence of 5.7 percent [1]. In two separate smaller surveys, 39% of people reported experiencing noises in the head or ears [2], [3]. Exposure to damaging sound is a leading cause of tinnitus in young people [4] and the second most common cause in older adults [1]. Notably, chronic tinnitus is reported by 35–50% of people with signs of noise-induced hearing loss [5]. Auditory damage not only reduces afferent input [6], but also appears to stimulate multiple compensatory central changes [7]. This compensation, or perhaps overcompensation, might produce the sensation of sound where none exists, i.e., tinnitus. Down regulation of inhibition has been proposed as a compensatory mechanism. After damaging acoustic exposure, down regulation of both γ-amino butyric acid (GABA) and glycine (Gly) transmission have been suggested as likely substrates [8]. In agreement with this hypothesis, facilitating inhibitory neurotransmission has been shown to reduce or eliminate tinnitus in animal models [9], [10], [11]. However, given the tightly regulated balance between inhibition and excitation in the central auditory system [12], [13], the potential contribution of excitatory neurotransmission to tinnitus should also be examined [14], [15], [16].

The dorsal cochlear nucleus (DCN), and more recently, the cerebellar paraflocculus (PFL), have been implicated in the pathophysiology of tinnitus [17], [18], [19], [20]. There are striking similarities between the circuitry of the DCN and the cerebellar cortex [21]. The unipolar brush cell (UBC) is found in both structures [22], [23]. The highest density of UBCs is in a small region, the “transition zone”, between the ventral PFL and the flocculus (FL) [24]. Acoustically responsive neurons in the PFL have been identified using electrophysiological methods [25], [26], [27]. Anatomical methods also indicate a descending acoustic input to the PFL from the secondary auditory cortex, via the pons, and a direct ascending input to the FL from the cochlea [25], [28], [29], [30].

Cerebellar UBCs receive glutamatergic inputs from mossy fibers and form glutamatergic synapses with their targets, granule cells and other UBCs. It has been hypothesized that UBCs may comprise a fan-out feed-forward excitatory network [31]. In the cerebellum, UBCs may also serve as variable-gain integrative amplifiers, controlled by GABAergic and glycinergic inputs from Golgi cells [32]. In the DCN, UBCs receive excitatory inputs from mossy fibers descending from the inferior colliculus and auditory cortex [33], thus potentially completing a positive feedback circuit. Considering both the DCN and ventral paraflocculus (vPFL), UBCs might contribute to signal processing through feed-forward and feed-back excitation. Recently it has been shown that UBCs in the DCN and PFL of rats show immunoreactivity (IR) for doublecortin (DCX) [34]. DCX is a microtubule associated protein that is expressed in newborn and migrating cells both developmentally and in the adult in regions of known neurogenesis, the dentate gyrus of the hippocampus and the subventricular zone [35], [36], [37], [38], [39]. Its expression in UBCs was unexpected, as there is no evidence for neurogenesis of UBCs in the adult animal (Baizer et al. unpublished observations). Expression of DCX in this cell population may indicate the involvement of these neurons in plastic processes [38] that affect signal gain. We have investigated that hypothesis with respect to the plastic changes underlying tinnitus.

While acute tinnitus typically occurs immediately after high-level sound exposure, chronic tinnitus often emerges gradually after an inducing event. For example, patients commonly report an insidious onset [40], and animal models have shown delayed onset from 5 to 90 days after high-level sound exposure [41], [42], [43]. The pathophysiological mechanisms responsible for tinnitus ultimately must account for the etiological features of the disorder. Upregulation of DCX in UBCs might be a component of the compensatory mechanism, and might help explain some of the etiological features. In addition, UBCs might provide a tractable target for therapeutic intervention. The purpose of the present research was to examine the potential role of UBCs and glutamatergic activity in chronic tinnitus, using an animal model.

## EXPERIMENT 1: Tinnitus-associated DCX elevation in UBCs of the DCN and vPFL

### Method

#### Subjects

8 male Long-Evans rats (Harlan, Indianapolis, IN, USA) 86 days old at the outset of the study, were individually housed and maintained at 25° C with a 12/12 h reversed light/dark schedule. Age at sacrifice for histology was 290–340 days. The animals were selected (selection described below) from a larger in-progress study investigating tinnitus. Three subjects were controls that had no history of noise-exposure. Five age-matched subjects had noise exposure and were selected from the larger study based on their strong behavioral evidence of tinnitus. Their age across the treatment sequence is summarized in Table 1. The experimental protocol was approved by the Laboratory Animal Care and Use Committee of Southern Illinois University School of Medicine.

**Table 1:** Treatment Groups.

Experiment 1		Age (days)	At Treatment	
Group (n)	Age at sound exposure	Age at training/testing	Age at sac/immuno-histology	
***Unexposed (3)***	120 (None, ABR only)	120–280	290–340	
***Exposed (5)*** *	120 (116 dB, 1 hr, & ABR)	120–280	290–340	

Experiment 2		Age (days)	At Treatment	
Group (n)	Age at sound exposure	Age at pre-drug testing	Age at drug pump implant and testing	Age at post-drug washout testing
***Unexposed (8)***: ***No tinnitus no drug***	88–91 (None, ABR only)	150–180	181–195 (No pump/no drug)	198–225
***Unexposed (8)***: ***No tinnitus agonist infusion***	88–91 (None, ABR only)	150–180	181–195 (AMPA + NMDA)	198–225
***Exposed (8)***: ***Weak tinnitus*** ** ***agonist infusion***	88–91 (120dB, 1 hr, & ABR)	150–180	181–195 (AMPA + NMDA)	198–225
***Exposed (8)***: ***Prominent tinnitus*** ** ***antagonist infusion***	88–91 (120dB, 1 hr, & ABR)	150–180	181–195 (D-AP5 + CNQX)	198–225
***Exposed (8)***: ***Prominent tinnitus*** ** ***no drug***	88–91 (120dB, 1 hr, & ABR)	150–180	181–195 (No pump/no drug)	198–225

* immunohistochemistry data unavailable for one animal.

** See text for definition.

#### Tinnitus Induction

At approximately 120 days old, 5 animals were unilaterally exposed once for 1 hr, to octave-band noise, with a peak level of 116 dB (SPL, re 20 µPa) centered at 17 kHz. The exposure was done during the initial stage of behavioral training. The animals were anesthetized with a ketamine-xylazine mixture (50 mg/kg and 9 mg/kg, respectively), placed in a modified stereotaxic head frame, and a speculum fitted with a speaker driver (Realistic 40–1398, Tandy Corp, Ft. Worth, TX, USA) was inserted into one ear canal. The contralateral canal was obstructed with a silastic ear plug filled with acoustic foam. This exposure, the methodological details of which are described elsewhere [44], [45], has been sufficient to produce tinnitus in at least 50 percent of rats within 3 months of exposure, and to temporarily elevate auditory brainstem evoked response (ABR) thresholds, 30–50 dB in the exposed ear, while leaving the contralateral ear unaffected. The 5 exposed animals in the present experiment were selected from the larger ongoing experiment, on the basis of their 20 kHz psychophysical performance demonstrating strong behavioral evidence of tinnitus. The 3 control animals were unexposed and were randomly selected from unexposed animals in the larger experiment.

#### Hearing Thresholds

ABR thresholds were obtained immediately before and after exposure, and at the conclusion of psychophysical testing. Unexposed animals were similarly tested without an immediate post-exposure measurement. ABR measurements were obtained with an IHS Smart EP System, running IHS High Frequency Software (v. 4.1) and using IHS high frequency transducers (Intelligent Hearing Systems, Miami, FL, USA). Acoustic stimuli were presented directly to the entrance of the ear canal. Stainless steel needle electrodes were placed subcutaneously at the vertex and over the bullae with a reference electrode at the occiput. ABR thresholds were obtained for 5 msec duration tone bursts presented at a rate of 50/sec. Tone bursts were gated using an exact Blackman envelope (2.5 msec rise/decay, 0 msec plateau) and presented in decreasing intensity series, beginning with levels that elicited distinct evoked potentials. Threshold was determined by the lowest intensity that produced visually distinct evoked waveforms within a 12 msec peri-stimulus window. Evoked potentials (X 200,000) were averaged over 512 sweeps.

#### Calibration

Sound levels throughout both experiments were calibrated using a Bruel & Kjaer Pulse sound measurement system (Pulse 13.1 software), equipped with a 3560C high-frequency module. Exposure levels and ABR levels were determined using a 4138 pressure-field microphone (Brüel & Kjaer, Norcross, GA, USA) coupled to the output transducers using rubber tubing with internal dimensions approximately matching that of an adult rat external auditory canal. Linear sound level determination was possible between 5 and 140 dB (SPL re 20 µPa), and from 5 Hz to 104 kHz. Sound levels in the behavioral test chambers were calibrated using a B&K 4191 open-field microphone. All levels reported are unweighted.

#### Tinnitus Assessment

Tinnitus was determined using a psychophysical procedure demonstrated to be sensitive to tinnitus and described in detail elsewhere [44], [45]. Briefly, an operant conditioned-suppression procedure was used to characterize the animals' perception of tones and silent periods presented in the context of ambient low level (60 dB, SPL) broad-band noise (BBN). Animals were tested daily in operant test chambers (Lafayette Instruments, Mod. 80001, Lafayette, IN, USA) equipped with lid-mounted speakers, metal floor grids and wall-mounted operant levers and food pellet dispensers. The animals were required to discriminate between the presence and absence of sound when tested with a variety of sounds of different composition, frequency, and level. Discrimination behavior was reflected in lever pressing for a food pellet reward. Absence of the ambient BBN noise, hence forth referred to as Speaker-off periods (1 min duration), acquired significance for the animals because lever pressing during these periods led to a foot shock at the end of the period (0.5 mA, 1 sec duration). While the features of tinnitus in rats (and humans) cannot be directly known, under no circumstance can tinnitus sound like silence. Consequently, any sensation experienced during Speaker-off test periods (i.e. silence or tinnitus) would be associated with foot shock. Foot shock could be avoided by not lever pressing (i.e., suppression). Lever pressing never led to foot shocks except during the speaker off (‘silent’ periods). The behavior of interest was lever pressing during randomly inserted probe tones (1 min duration) that substituted for some of the speaker-off presentations. Each daily test session contained 10 randomly inserted, non-contiguous, probe tone presentations with background BBN off. Two of the ten were always Speaker-off periods. The remaining 8 were of a randomly-selected tone or noise, with presentation levels between 30 dB and 90 dB SPL. The decision task for the animals was to discriminate between the test stimulus and Speaker off. Lever pressing was quantified using a relative rate measure, the suppression ratio (R). R was determined as a running measure for successive 1-min segments of each session using the formula R  =  B/(A+B), where A was the number of lever presses in the preceding 1-min segment and B the number of lever presses in the current 1-min segment. R can vary between 0 and 1. A value of 0 is attained when lever pressing in the current minute is 0, a value of 0.5 when lever pressing in the current minute is equal to that of the previous minute, and a value of 1 when lever pressing in the previous minute is zero. R provided a running index of behavior, in 1-min epochs, and enabled a quantitative comparison between subjects as well as unbiased compilation of group data. R is a useful index of perceptual performance since it is very sensitive to short-term behavioral effects, such as those produced by sensory events, but it is very insensitive to gradual behavioral effects, such as those produced by changes in motivational status, for example, satiation. In the context of the present procedure, it was expected that exposed rats with tinnitus would have lower R-values than unexposed rats, when tested with stimuli that resembled their tinnitus. All test sessions were 60 min in duration. Further details of the psychophysical procedure are available in an open-source document [45].

#### Immunohistochemistry

At the conclusion of psychophysical testing, following final ABR assessment, subjects were given a lethal intraperitoneal dose of a commercial euthanasia agent (Euthasol, Virbac, Ft. Worth, TX), and perfused transcardially with 0.9% normal saline followed by 4% paraformaldehyde in 0.1 M phosphate buffer at room temperature (21°C). The brain was extracted, taking care to preserve the PFL, and stored in 4% paraformaldehyde at 4°C for 24 hr. Prior to sectioning, the brains were cryoprotected in 20% sucrose for 24 h. Coronal sections (50 µm), encompassing the DCN and PFL, were floated in phosphate buffer and stored at 4°C in cryopreservative (30% glycerol, 30% ethylene glycol, 40% PBS) until processing.

All processing was performed using free-floating sections. Sections were removed from the cryopreservative and rinsed in PBS. Non-specific binding of primary antibodies was blocked by incubating the sections in a solution of 1% bovine serum albumin (BSA, Sigma, St. Louis, MO, USA), 1.5% normal horse serum (NHS, Vector laboratories, Burlingame, CA, USA) and 0.1% TritonX-100 (TX) in PBS for 30 min. A primary antibody against doublecortin (DCX; Santa Cruz Biotechnology, SCBT, catalogue # sc-8066, goat polyclonal, 1:500) was added and the sections incubated overnight at 4°C on a tissue rocker. Sections were then rinsed and incubated in an anti-goat biotinylated secondary antibody (Vector Laboratories, Burlingame, CA, USA, following manufacturer's instructions); further processing followed the Vector ABC method using a Vectastain kit. More specifically, immunoreactivity was visualized using the glucose oxidase modification of the diaminobenzidine (DAB) method [46], [47]. Sections were rinsed and mounted on Fisher “Superfrost” polarized slides (Fisher Scientific, Pittsburgh, PA, USA). The slides were dehydrated in graded concentrations of ethanol, cleared in xylene or Histosol (National Diagnostics, Atlanta, GA, USA) and coverslipped with Depex (Electron Microscopy Sciences, Hatfield, PA, USA), or Permount (Fisher Scientific). The sections were examined with a Leitz Dialux 20 light microscope, and digital images were captured with a SPOT Insight Color Mosaic camera (1200×1600 pixels).

#### Data analysis: Immunohistochemistry. DCX in UBCs

**Figure 1:**
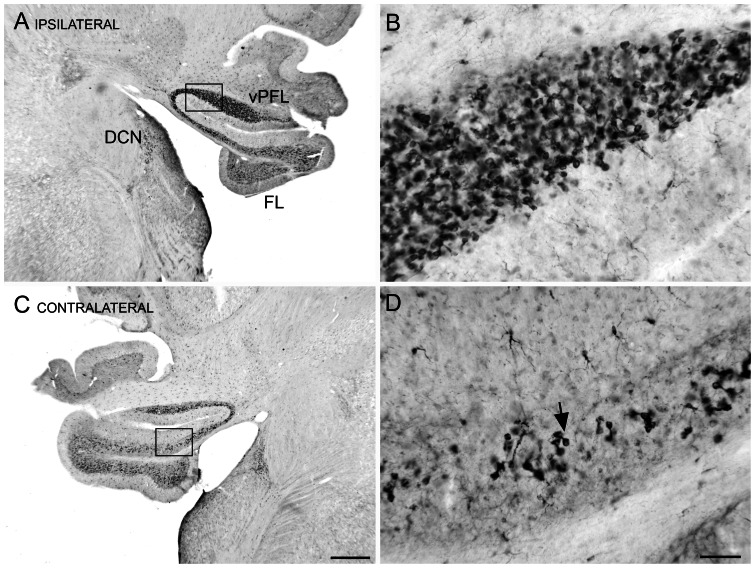
Doublecortin (DCX) immunoreactivity (IR) in UBCs of the DCN and cerebellum ipsilateral to the exposure. **A.** Photomicrograph through the brainstem and cerebellum showing DCX-IR neurons. The rectangle shows the location of the higher magnification photomicrograph in B. **B.** DCX-IR in UBCs of the vPFL. **C.** DCX-IR neurons in the vPFL, FL and DCN on the side contralateral to the exposure. The rectangle shows the location of the photomicrograph in D. Scale bar 500 µm. **D.** DCX-IR neurons are UBCs, example at arrow. Scale bar 50 µm. Abbreviations: DCN, dorsal cochlear nucleus; FL, flocculus; UBC, unipolar brush cell vPFL. ventral paraflocculus.

Brain section images at 2x magnification, containing both the DCN and mid-rostro-caudal vPFL (Fig. 1), were imported into Image J (ver. 1.44p, http://imagej.nih.gov/ij) for analysis. Sections from one exposed animal were of poor quality and were discarded. Sections from the remaining 7 animals (4 exposed and 3 unexposed) were analyzed. Each image was converted to 32-bit grayscale, and the areas of interest (AOI), i.e., the DCN or vPFL/FL (Fig. 1), were isolated from background. Image J area measurement tools were calibrated using a physical standard photographed at settings used for brain micrography. Black/white thresholding was visually adjusted for each image to distinguish DCX IR particles from background. Setting particle image thresholds individually for each micrograph compensated for variation in image brightness. The Image J particle tool was used to determine both the number of discrete IR particles and the area immunostained in each micrograph. Care was taken to exclude blemishes and confounding features such as the high-contrast borders of the brain sections. The numeric results were exported to Excel spreadsheets (Professional Edition, 2007, Microsoft, Redmond, WA, USA) for determination of IR area and particle density/AOI. Comparison between exposed and unexposed DCN and vPFL were made using uncorrected independent t tests. IR area was a more discriminant measure of density than particles counts. Immunostained particles were of irregular shape, and particle counts were negatively biased by overlapping and adjacent particles (most pronounced in the vPFL), making particle separation unreliable (Fig. 1). Characterizing DCX IR in terms of percent area appeared to be the least biased way to present the results. Cell density data were suspect for large areas with high levels of DCX IR, such as in the cerebellum. In the cerebellum many overlapping cells were stained, making unbiased and automated cell partitioning impossible. With the validity of cell counting in question, density estimates based on cell counting would also be in question. Tinkering with antibody concentrations in order to reduce IR was not considered because that would have made DCX detection in sparse areas, such as the DCN, more problematic. It also would have altered procedural parameters from those of an established method [34]. Therefore no attempt was made to directly estimate the number of DCX-IR neurons. IR area was assumed to be an unbiased estimator of DCX IR cell density.

#### Data analysis: Tinnitus psychophysics

Behavioral data were entered into spreadsheets. For inclusion in statistical analysis, individual-subject data sets had to pass two quality criteria: (a) There had to be a minimum of 200 total lever presses in the session, and (b) the average suppression ratio during non-test periods (i.e., during background (BBN) had to be at least 0.4. These conjoint criteria insured that an adequate number of lever presses were distributed across the 1-hr test session to make the response to individual test-tone presentations reliable. Descriptive and inferential statistical analyses and graphic depictions were done using Excel. Between group differences were analyzed using mixed-model ANOVAs applied to all data, excluding “speaker off” results.

Experiment 1: Results

#### Exposure and hearing threshold

**Figure 2:**
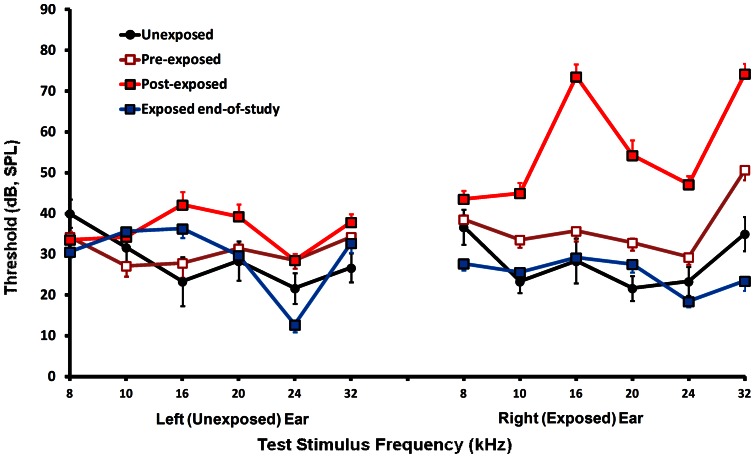
Auditory brainstem response (ABR) group-average thresholds before and after high-level sound exposure. Unexposed rats are included for comparison. Post-exposure thresholds, obtained immediately after right ear exposure, show temporary right (exposed) ear elevation. End-of-study thresholds, obtained at the conclusion of psychophysical testing, show a recovery of exposed animals to normal threshold levels. Error bars in this and other figures indicates the standard error of the mean.

As reported in previous experiments [44], a temporary ABR threshold elevation was evident in the exposed ear immediately after exposure. Thresholds resolved to unexposed-control levels at the conclusion of psychophysical testing, approximately 140 days after exposure (Fig. 2). Thresholds of unexposed ears were not affected.

#### Exposure and psychophysical evidence for tinnitus

**Figure 3:**
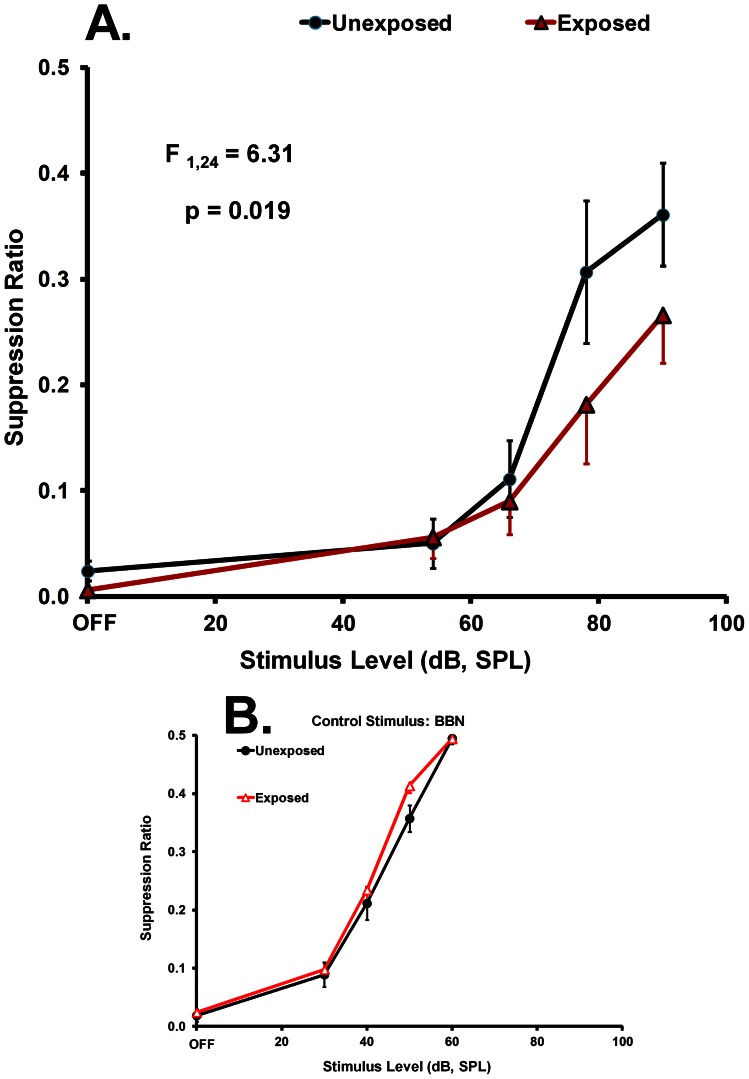
Group average psychophysical discrimination functions of exposed and unexposed animals in Experiment 1. The suppression ratio, plotted on the y-axis, indicates relative lever press rate. Sound level, plotted on the x-axis, indicates the ambient level in the 1-min test periods that substitute for background noise. The functions slope upward from speaker OFF because food is obtained for lever pressing when the sound is on, but a foot shock is delivered at the end of speaker OFF periods when speaker-off lever pressing is above a criterion level. Panel A shows the results when tested with the diagnostic stimulus of 20 kHz tones. Panel B shows the results when tested with the non-diagnostic stimulus of broad-band noise (BBN). Hearing level is comparable in both exposed (triangular data points) and unexposed (circular data points) animals, as indicated by their BBN performance. The down-shift of the exposed rats' function when tested with 20 kHz shows greater suppression to sound resembling their sensation during speaker off periods, i.e., tinnitus. Statistically significant separation of the functions above speaker-off is indicated by repeated-measures ANOVA in Panel A.

In this paradigm, tinnitus is typically indicated by a discrimination function downshift to one or more of the test tones. The downshift, with respect to non-tinnitus controls, indicates that the tone in question resembled the auditory percept during speaker off periods. These results were obtained when the exposed animals were tested with 20 kHz tones (Fig. 3A; summary statistics appear in the figure; statistical analysis described in 1 Methods). In comparison, discrimination of other stimuli, such as BBN, did not differentiate exposed from unexposed animals (Fig. 3B).

#### Tinnitus and UBC immunoreactivity

**Figure 4:**
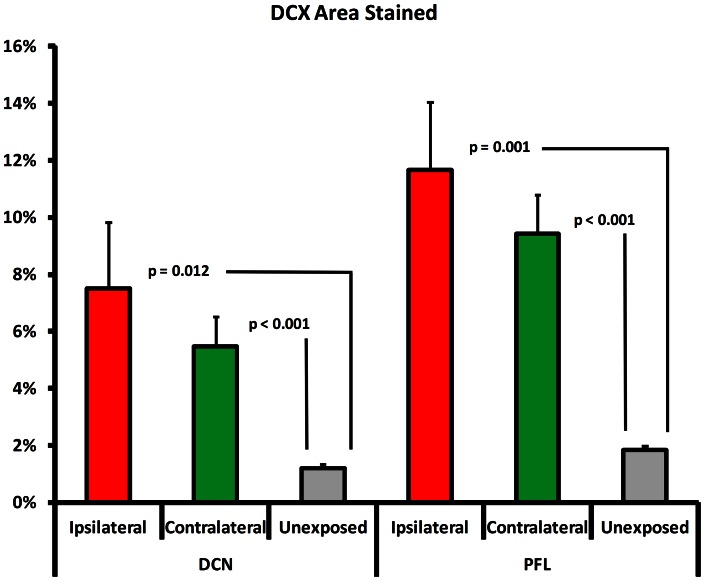
Doublecortin (DCX) immunoreactivity in the ipsilateral and contralateral DCN and PFL of exposed rats, and unexposed rats. Both Ipsilateral and contralateral DCN and PFL showed significant elevation (independent t tests) above that of unexposed control animals, with higher ipsilateral levels.

The percent area positively stained for DCX IR was bilaterally elevated in the DCN and PFL of exposed rats compared to unexposed controls (Fig. 4; significance values appear in the figure). Ipsilateral DCX IR was greater than contralateral, and overall PFL IR was higher than DCN IR. DCX marked cells were evident throughout the vPFL, but in the DCN they clustered primarily in the dorsal cap (Fig. 1 is representative).

### Experiment 1: Conclusion

DCX IR was elevated in both the DCN and PFL of rats showing psychophysical evidence of chronic tinnitus. In both the DCN and PFL the elevation was evident bilaterally, but was more pronounced ipsilateral to the exposed ear (Fig. 4). For exposed animals, DCX IR area was greater in the PFL than in the DCN. Increased DCX IR reflects increased levels in UBCs but cannot discriminate between increased DCX expression within a positive cell or an increase in the number of cells expressing DCX. In either case, the increase in total area with +DCX IR may reflect neuroplastic alterations in cell function associated with tinnitus. Manipulating glutamatergic activity within central neural structures containing UBCs would be a logical next step investigating the potential role played by these areas related to tinnitus.

## EXPERIMENT 2: The effect of glutamatergic modulation in the PFL on tinnitus

If UBC plasticity contributes to post-traumatic auditory compensation and tinnitus, then local manipulation of glutamatergic transmission in either the PFL or DCN should modulate tinnitus. The objective of Experiment 2 was to pharmacologically manipulate glutamatergic transmission in the PFL of animals with and without tinnitus while psychophysically quantifying their tinnitus.

### Method

#### Subjects

42 male Long-Evans rats (Harlan, Indianapolis, IN, USA) approximately 90 days old at the outset of the study, were individually housed and maintained at 25°C with a 12/12 h reversed light/dark schedule. Age at the conclusion of psychophysical testing was 225 days. The experimental protocol was approved by the Laboratory Animal Care and Use Committee of Southern Illinois University School of Medicine.

#### Procedure: Subgroups and treatments

Tinnitus was induced and quantified using methods identical to those in Experiment 1. Twenty-six randomly selected rats were unilaterally exposed to high-level sound for 1 hr, while 16 were unexposed. At exposure, the animals were 88 to 91 days old. As in Experiment 1, all animals, exposed and unexposed, were trained and tested in parallel, using the previously described psychophysical procedure. After tinnitus was characterized, subgroups were formed based on psychophysical performance (summarized in Table 1).

The unexposed animals were divided into two subgroups of 8 each, with equivalent psychophysical performance profiles. One subgroup was randomly assigned to receive a cocktail of glutamatergic agonists delivered chronically for 14 days to the right PFL (drugs and delivery method described below). The objective was to induce tinnitus-like symptoms solely through administration of glutamatergic agonists localized to a zone of high UBC density, as indicated by DCX IR. This drug treated subgroup was labelled “unexposed agonist.” The second unexposed subgroup remained untreated and was labelled “unexposed no-drug.” The unexposed no-drug group provided a negative control, i.e., no tinnitus and untreated.

Two exposed rats that failed to meet behavioral performance criteria were dropped from psychophysical testing and used later to histologically assess pump function and extent of tissue infusion. The remaining 24 exposed animals were divided into three subgroups of 8 each. Subgroup assignment was determined by 20 kHz psychophysical performance, i.e., the diagnostic probe tone that reflected tinnitus. Sixteen of these 24 with the lowest mean suppression ratio during 20 kHz probe tone periods were designated “prominent tinnitus.” These sixteen rats were then randomly divided into two subgroups. These two subgroups were not significantly different from one another but they were each significantly different from the unexposed controls (statistical analysis in Results). The remaining 8 exposed animals, i.e., those with the highest suppression ratios during 20 kHz probe tone periods, were designated “weak tinnitus.” They were not significantly different from unexposed controls (statistical analysis in Results).

The exposed subgroup with weak tinnitus was assigned to receive the glutamatergic agonist cocktail delivered chronically for 14 days to the PFL ipsilateral to the sound exposed (right) ear. The objective was to enhance their weak tinnitus via local administration of glutamatergic agonists. Treatment of this “exposed-agonist” subgroup therefore paralleled that of the unexposed-agonist subgroup.

One of the two “prominent tinnitus” subgroups received a cocktail of glutamatergic antagonists, chronically delivered for 14 days to their ipsilateral PFL. The objective was to attenuate their tinnitus and for purposes of analysis they were labeled “exposed antagonist.” The second “prominent tinnitus” subgroup did not receive drugs and as such they were labeled “exposed no-drug.” This subgroup served as a positive control, i.e., significant tinnitus without drug modulation. Vehicle-only control groups were not run because vehicle effects would be evident when comparing the exposed agonist and exposed antagonist groups to one another and to untreated groups.

#### Procedure: Drug delivery

**Figure 5:**
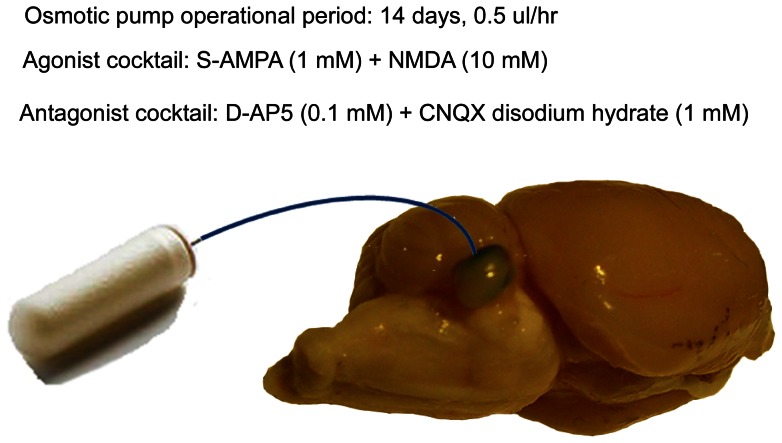
Schematic of the osmotic pump delivery of drugs in Experiment 2. The pump body was subcutaneously implanted in the dorso-caudal neck region. The catheter terminated dorsal to the ipsilateral PFL, anchored to a 1 mm diameter skull opening. Infusion volume and rate are shown.

The drug delivery method was chosen to continuously infuse a circumscribed brain area of high UBC density with glutamatergic agonists or antagonists for the time required to complete psychophysical testing (i.e., 2 weeks). Sterile drug solutions were loaded into osmotic mini pumps (Alzet Model 2002, Durect Corp., Cupertino, CA, USA). Each pump delivered 0.5 µl per hour for a minimum of 14 days. Under aseptic conditions, each drug-treated animal received a single pump, placed subcutaneously in the caudal neck region while under isoflurane anesthesia (2% at 0.8 L/min). The pump was anchored to subdermal muscle and connective tissue using a nylon suture. Pump output was delivered via a length of PE-50 catheter tubing, the end of which fit tightly into a 1 mm craniotomy made in the subarcuate fossa, above the dorsal aspect of the right PFL. In exposed animals this was ipsilateral to their exposed ear (Fig. 5). A flare formed at the distal catheter tip made a tight seal within the craniotomy, and the catheter was further sealed to the skull surface using bone wax and thermal polyethylene adhesive (Best Stik, FPC Corp., Wauconda, IL, USA). The skin incision was closed with wound clips and the animals were allowed to recover for 48 hrs before psychophysical testing resumed. Fifteen days after implant, the animals were again anesthetized and their catheters and pumps were removed. At the time of removal, both the position and patency of the catheters was confirmed. In addition, the pumps were inspected to confirm that their drug reservoirs were empty. Six of 24 animals had pump-catheter implants failing to meet placement/patency criteria, two from each drug-treated subgroup (unexposed, exposed “weak tinnitus”, exposed “prominent tinnitus”). All data from these animals were excluded from analysis.

**Figure 6:**
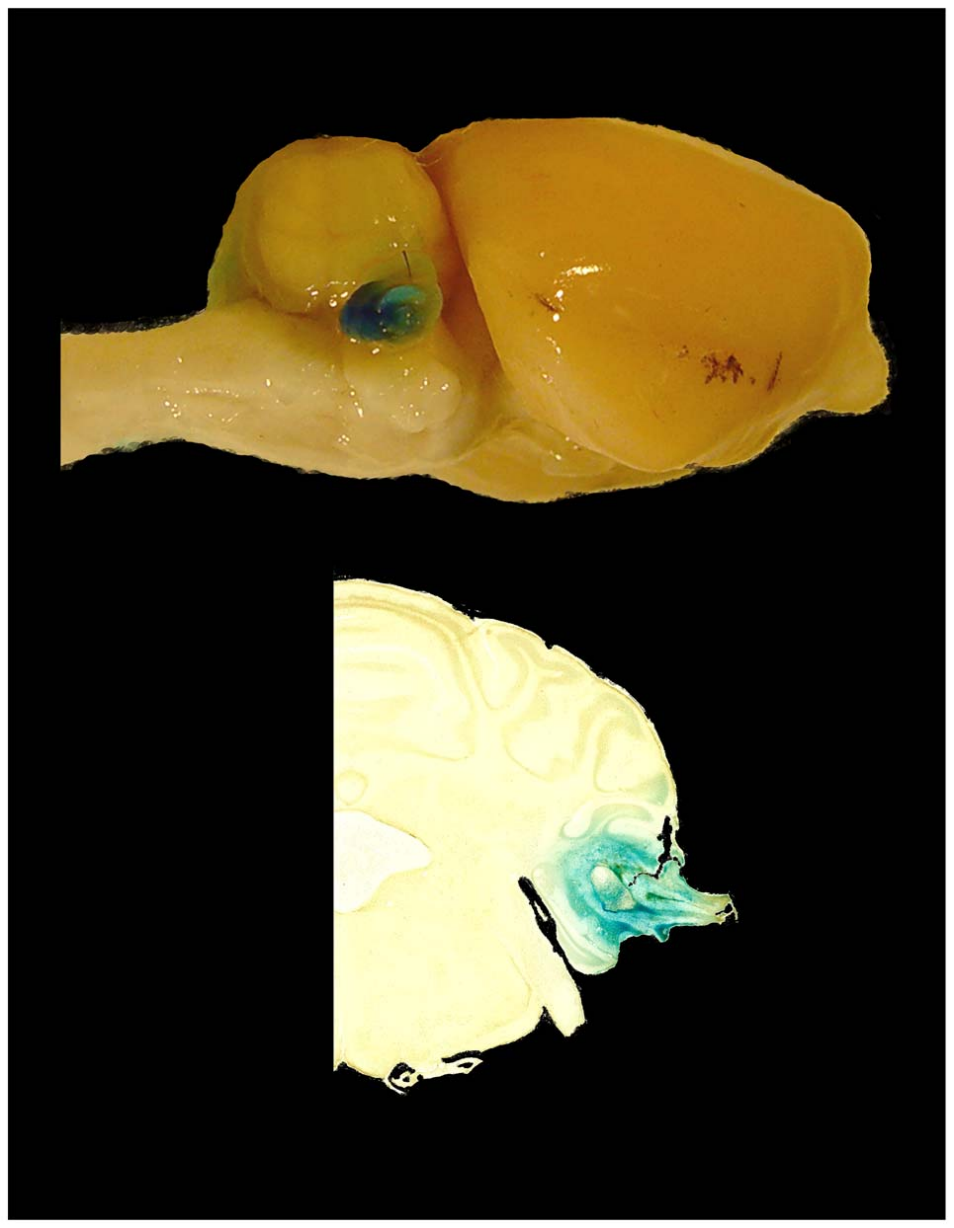
Drug diffusion as indicated by methylene blue. Two rats were implanted with pumps filled with 0.25% methylene blue to indicate the diffusion field of pump-delivered drugs. The diffusion coefficient of methylene blue was 85% that of the drug cocktail average diffusion coefficient. The top panel shows the macroscopic diffusion of methylene blue over the surface of the PFL. The lower panel shows deep tissue penetration in a coronal section of the PFL. Methylene blue infused and penetrated the PFL up to its stalk, but did not extend into brainstem areas encompassing the cochlear nucleus.

The two animals removed at the study outset for poor behavioral performance were implanted with osmotic mini pumps identical to those described. The pumps were filled with 0.25% methylene blue in normal saline. Methylene blue was chosen because its molecular weight (374) is reasonably close to the average molecular weight of the glutamatergic drugs used (mean of 201). As determined by classic diffusion equations [48], the diffusion coefficient of methylene blue is 85 percent that of the glutamatergic cocktails. After 14 days the pumps were removed and the animals were transcardially perfused with normal saline followed by 4% paraformaldehyde in PBS. The surface of the PFL and brainstem were photographed and the brains prepared for frozen sections using the procedure described in Experiment 1. The histological sections and macro photographs were used to confirm the drug delivery and extent of diffusion within the subarcuate fossa (Fig. 6).

#### Procedure: Drugs and doses

The glutamatergic antagonist cocktail was made from an equal-volume mixture of the NMDA-receptor (N-methyl-d-aspartic acid) antagonist, D-AP5 (D(−)-2-Amino-5-phosphonopentanoic acid, Tocris Bioscience, Boston, MA, USA), 0.1 mM, and the AMPA-receptor (α-amino-3-hydroxy-5-methylisoxazole-4-propionic acid) antagonist, CNQX (6-cyano-7-nitroquinoxaline-2,3-dione disodium salt hydrate, Tocris Bioscience, Boston, MA, USA), 0.1 mM, dissolved in sterile saline. Drug solution pH was approximately 7. The objective of the antagonist cocktail was to locally block glutamatergic NMDA and AMPA receptors in a cerebellar zone of high UBC density.

The glutamatergic agonist cocktail was made from an equal-volume mixture of NMDA, 10 mM (Tocris Bioscience, Boston, MA, USA), and S-AMPA, 1 mM (Tocris Bioscience, Boston, MA, USA) dissolved in sterile saline. Drug solution pH was approximately 7. The objective of the agonist cocktail was to locally activate ionotropic glutamatergic NMDA and AMPA receptors in a cerebellar zone of high UBC density.

Agonist and antagonist concentrations were determined from a survey of contemporary literature, whenever possible relying on studies using direct *in vivo* injection. Although data on continuous central microinfusion of glutamatergic compounds were not available, Li et al. found that repeated direct micro-injection of 1–2 mM AMPA, and 1–10 mM NMDA, to rat hypothalamus, produced significant behavioral effects without histological evidence of tissue damage [49]. Vehicle (sterile normal saline) control infusions were not employed for several reasons: (a) specific vehicle effects were unlikely; (b) nonspecific vehicle effects, such as fluid-volume tissue disturbance would be equivalent in both the agonist- and antagonist-treated groups, while agonist – antagonist effects would be in opposite directions; (c) adding a vehicle control for each of the three drug treatment groups would have significantly increased both the complexity of the experimental design and the number of required animals.

### Experiment 2: Results

#### Glutamatergic blockade

**Figure 7:**
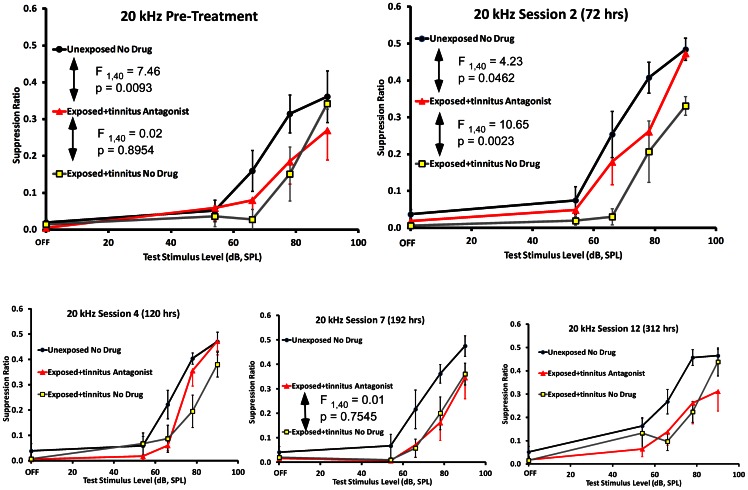
Experiment 2, glutamatergic antagonist effects on exposed rats with tinnitus. Glutamatergic antagonists were delivered to the ipsilateral PFL of exposed rats with significant evidence of tinnitus (triangular data points): Unexposed group results are depicted as circular data points and exposed non-drug animals as square data points. Pre-drug data are shown in the upper left panel. Exposed pre-drug animals were significantly downshifted from unexposed controls and were no different than exposed no-drug animals with tinnitus (repeated-measures F statistics shown in panel). After 72 hrs of antagonist cocktail, the treated animals were significantly up-shifted from the untreated exposed animals, therefore showing attenuation of tinnitus. Antagonist amelioration of tinnitus gradually waned over subsequent treatment days (bottom panels, left to right), returning to pre-treatment strength at 192 hrs.

The antagonist cocktail of D-AP5 and CNQX was successfully administered to the ipsilateral PFL of 6 exposed rats that displayed significant pre-drug psychophysical evidence of tinnitus. The antagonist produced a partial but significant attenuation of tinnitus after 72 hrs of infusion (Fig. 7, top right panel). This therapeutic effect gradually washed out, becoming non-significant 192 hrs after the start of infusion (Fig. 7, lower middle panel). Therefore ionotropic glutamatergic blockade in the ipsilateral PFL temporarily reduced, but did not eliminate, tinnitus.

#### Glutamatergic activation

**Figure 8:**
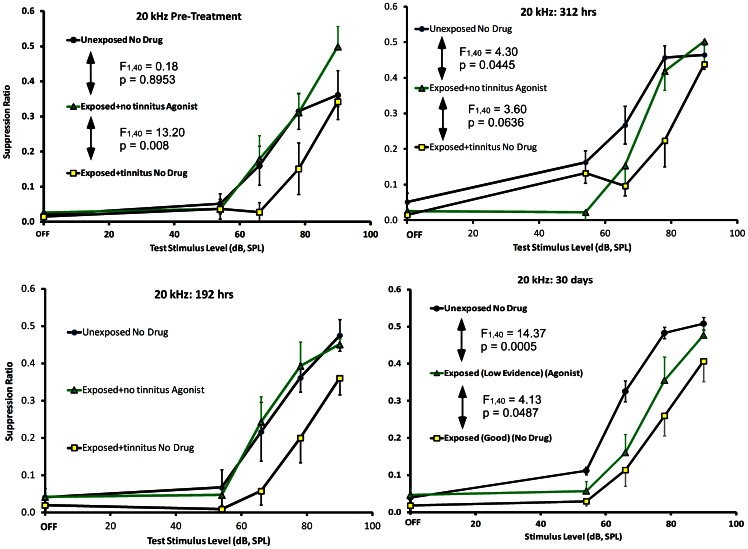
Experiment 2 glutamatergic agonist effect on exposed rats with no evidence of tinnitus (triangular data points). The results of unexposed and exposed control groups are depicted as in Figure 7. Pre-drug data are shown in the upper left panel. The agonist cocktail induced significant tinnitus in the exposed no-tinnitus animals, indicated by a function down-shift (upper right panel) after 300 hrs of treatment, but their tinnitus did not attain the same level as the exposed group with tinnitus (F statistics in panel). Nevertheless, the agonist-induced tinnitus persisted for at least 30 days post drug (lower right panel), at which time testing was discontinued.

The agonist cocktail of NMDA+AMPA was successfully delivered to the ipsilateral PFL of 6 exposed rats with weak pre-drug evidence of tinnitus. The agonist cocktail exacerbated their tinnitus, with the greatest effect evident 312 hrs into the infusion period, which was near the end of the pump operational period (Fig. 8, top right panel). The agonist-induced exacerbation was long lasting, and was still evident 30 days after discontinuation of the infusion period (Fig. 8, lower panels). One interpretation would be that the high-level sound exposure, months prior, produced sub-threshold alterations in brain function that were subsequently pushed over threshold by glutamatergic activation.

**Figure 9:**
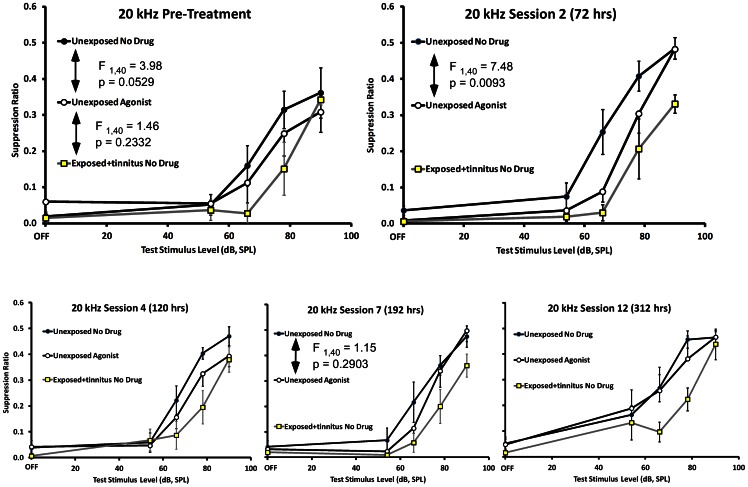
Experiment 2 glutamatergic agonist effect on unexposed rats with no evidence of tinnitus. The glutamatergic agonist cocktail was delivered to the right PFL of unexposed rats with no evidence of tinnitus (open circular data points). Unexposed and exposed control groups are depicted as in Figure 7. Pre-drug data are shown in the upper left panel. The agonist cocktail induced a significant tinnitus-like downshift in the function of unexposed animals (upper right panel) after 72 hrs, although, as before, their tinnitus did not attain the same level as the exposed group with tinnitus (F statistics in panel). This tinnitus-like induction gradually washed out over the course of treatment (lower panels, left to right).

The agonist cocktail of NMDA+AMPA was also successfully delivered unilaterally to the PFL of 6 unexposed rats with no evidence of tinnitus. The agonist cocktail produced tinnitus-like symptoms, with the greatest effect evident 72 hrs into the infusion period (Fig. 9, top right panel). This effect was temporary, disappearing 192 hrs into the infusion period (Fig. 9, lower middle panel).

#### Indication of drug delivery

Infusing methylene blue as a substitute for the drug solution permitted the extent of drug diffusion to be estimated. As indicated by methylene blue staining, the infusion field encompassed the entire PFL (Fig. 6, top panel). Histological sections showed the infusion field invaded the stalk of the PFL and its base, but did not enter the brainstem or other cerebellar areas (Fig. 6, bottom panel). Based on these data, and the similar diffusion coefficients of methylene blue and the drug cocktails, the drug delivery field was estimated to encompass the ipsilateral PFL, but not to extend significantly into surrounding tissue.

### Experiment 2: Conclusion

Glutamatergic agonists and antagonists unilaterally localized to the PFL, were shown to modulate tinnitus or tinnitus-like behavior. Rats with confirmed evidence of tinnitus showed a partial and transient decrease in their tinnitus when challenged with combined NMDA and AMPA blockade. Conversely, normal-hearing rats, when challenged with combined NMDA and AMPA agonists, displayed reversible tinnitus-like psychophysical behavior. Animals that did not develop significant tinnitus after having been exposed to a tinnitus-inducing sound, when treated with the glutamatergic agonists, showed significantly increased tinnitus that persisted for at least a month after treatment. In summary, modulation of glutamatergic activity in the PFL was effective in modulating psychophysically-defined tinnitus in a rat model.

## General Discussion

A contemporary conceptual view of the cerebellum is that of an adaptive control engine [50]. This concept is typically presented in the context of motor control and learning. However, virtually all experiments that support adaptive control also demonstrate a modified reaction to sensory input. Tinnitus could be placed within that framework, when considered as an altered auditory response following acoustic trauma.

UBCs are excitatory glutamatergic interneurons found in the DCN and PFL [31]. They have been hypothesized to comprise tunable local amplifier circuits in the cerebellum [32]. Cerebellar UBCs have been shown to be controlled by a balance of excitatory input from mossy fibers and inhibitory inputs from Golgi cells [32]. Interestingly, subclasses of UBC's may be influenced quite differently by their major input, mossy fibers. One class increases spontaneous activity when mossy fibers are turned off [32]. This inverter function could translate reduced afferent activity, such as that following acoustic trauma, into elevated output. *In vivo* single-unit recording has shown that UBCs in rats have a relatively high spontaneous activity level of 10 to 50 spikes per second, and a narrow distribution of inter-spike intervals [51]. This activity profile, i.e., elevated spontaneous activity with synchronous discharge, when encountered in auditory areas, has been hypothesized to underpin tinnitus [52]. Although the correspondence of UBC spontaneous activity to this profile might be coincidental, it should not be discounted entirely, even though classical models of the cerebellum assign output exclusively to Purkinje cells. Purkinje cells can be influenced by UBCs via granule cell parallel fiber connections.

Recently UBCs in the vPFL have been shown to express DCX, previously thought to be a marker of recent mitosis and cell migration [34]. In Experiment 1, using a method identical to that of Manohar et al. [34], DCX IR was shown to be significantly elevated, with respect to unexposed controls, in both the DCN and vPFL of rats with evidence of sound-trauma-induced chronic tinnitus. This evidence is consistent with a hypothesis of plastic up-regulation of DCX expression in this cell population induced by auditory deafferentation. If the up-regulation involved structural/metabolic changes, then delayed or gradual onset of chronic tinnitus would be expected. Turner, et al. [43] reported that rats exposed to tinnitus-inducing sound did not develop full evidence of chronic tinnitus until 3 months after exposure. The Oregon Tinnitus Archive reports that of the 844 individuals comprising Data Set 1, 50.9 percent reported gradual onset of more than one month, and of the 437 individuals comprising Data Set 2, 53.9 percent reported gradual onset of more than one month [40]. Therefore, the gradual onset of chronic tinnitus observed both experimentally and clinically, is congruent with a gradually emergent plasticity.

The results of Experiment 1 showed an association between UBC DCX level and tinnitus in an animal model. If increased DCX level in UBCs indicates a plastic upregulation that contributes to the perception of tinnitus, then a causal connection might be established by administration of glutamatergic agonists and antagonists to an area of high UBC density, such as the PFL: that is, glutamatergic agonists and antagonists should modulate tinnitus when delivered to the PFL. This was confirmed in Experiment 2. Combined NMDA and AMPA receptor blockade temporarily, but significantly, attenuated tinnitus with respect to untreated controls. Partial and temporary attenuation was not surprising given the method of drug delivery. A number of factors could account for the partial effect, even in the unlikely event that UBC plasticity was exclusively responsible for tinnitus. Although the delivery diffusion tests using methylene blue (Fig. 6) indicated that the drug very likely dispersed throughout the PFL, there was no evidence that it invaded tissue outside the subarcuate fossa. A substantial number of UBCs would therefore have been spared because they were located in areas uninfluenced by the antagonists (or agonists). Uninfluenced areas would have included the ipsilateral and contralateral DCN, as well as the contralateral PFL. Additionally, the drug diffusion gradient within the ipsilateral PFL could have left some local receptors unaffected. Although the drug delivery method had limitations, the obtained significant temporary tinnitus attenuation supported the hypothesis of a cerebellar glutamatergic tinnitus component to which UBCs could have contributed. Of course the contribution of other glutamatergic elements cannot be ruled out. Aside from UBCs, the potential contribution of granule cells should be considered. Granule cells are glutamatergic and are numerous in both the cerebellum and DCN [53]. Separating UBC from granule cell contribution is an objective for future research, although at present selective UBC agonists or antagonists are unavailable.

A question may be raised about not targeting the DCN for drug treatment, given the results of Experiment 1 showing DCX IR in the DCN of tinnitus animals. The DCN was not targeted because it would have been very difficult to deliver glutamatergic agents to the DCN *in vivo* without also affecting the ventral cochlear complex. Glutamatergic modulation of the ventral cochlear nucleus would have interfered with sensation from the ipsilateral ear, making interpretation of results difficult. Another reason for initially targeting the PFL and not the DCN was that Experiment 1 showed the greatest changes in DCX IR after acoustic trauma to be in the PFL, not the DCN. Finally, prior work has shown that unilateral and bilateral ablation of the DCN does not reduce or eliminate established acoustic trauma related tinnitus in an animal model [54].

A further question may be raised about the potential involvement of the DCN in mediating the drug effects obtained in the present study. Although use of the indicator methylene blue showed that the pump-delivered drugs did not extend beyond the PFL, very low levels may have nevertheless reached receptors in the DCN. Independent evidence suggests that if this happened it is unlikely to be responsible for the obtained drug effects, at least for the antagonists. As noted, DCN ablation experiments showed that surgical lesions of the DCN in animals with established tinnitus had no impact on their psychophysically defined tinnitus [54]. The interpretation was that once tinnitus is established, reduction of DCN output alone is not sufficient to modify the tinnitus, although the DCN might still serve as a necessary trigger zone for the initiation of tinnitus [45].

The present experiment cannot discriminate between the potentially distinct contributions of NMDA and AMPA receptor subtypes to tinnitus generation and modulation. It was deemed important in these initial experiments to confirm a cerebellar glutamatergic component. Since the relative contribution of NMDA and AMPA receptors to UBC network function, and tinnitus, is unknown, both receptor subtypes were targeted. For example, while long-term potentiation and depression typically depend upon the plasticity of NMDA receptors, in the cerebellum long-term depression might be mediated by an endocytotic removal of post-synaptic AMPA receptors from dendritic spines [47]. One model of long-term memory storage in the cerebellum hypothesizes that short-term plastic alterations at parallel-fiber Purkinje synapses are replaced by long-term alterations at parallel-fiber stellate/basket cell synapses [55]. Parallel-fiber stellate/basket cell synapses comprise both NMDA and AMPA receptors.

In Experiment 2 a glutamatergic agonist cocktail was administered to the PFL of unexposed control rats without tinnitus, as well as to the PFL of exposed rats with minimal evidence of tinnitus. The objective was to exacerbate nonsignificant tinnitus in exposed animals and to produce tinnitus-like symptoms in the normal controls by pharmacologically driving cerebellar UBCs. Within 72 hrs of administration, the AMPA-NMDA cocktail produced significant tinnitus-like behavior in normal control animals (Fig. 9). This effect was transient, and disappeared 192 hrs into the administration period. Several factors could account for the transience of the effect. AMPA stimulation of glutamate receptors *in vitro* is well-known to produce desensitization [56]. Cumulative desensitization could account for the limited duration of effect. Additionally, the cerebellum is rich in inhibitory feedback circuits, which could function to limit the excitation produced by exogenous agents. GABA-mediated recurrent and feed-forward inhibition has been well-characterized in cerebellar circuits [53]. Basket cells, for example, have been shown to strongly inhibit Purkinje cells, the efferents of cerebellar cortex, and as such they contribute to rate-limiting Purkinje output when the Purkinje cells are driven by glutamatergic inputs [53]. Direct application of NMDA to cerebellar Purkinje cells and associated interneurons *in vitro* has been reported to have complex effects, but generally mini-inhibitory post-synaptic potentials were increased in Purkinje cells [57]. Since Purkinje cell output is exclusively GABAergic, generalizing from these results, the net effect of NMDA release in the cerebellum would be to release extra-cerebellar targets from inhibition. Prediction of global effects from local circuit experiments, however, is fraught with hazard, since complex feedback interactions within the PFL and DCN make global generalization difficult. A simple hypothesis is that the immediate action of glutamate in the cerebellum would be to enhance cerebellar inhibition of efferent targets. This prediction derives from glutamatergic parallel fibers driving Purkinje cells, which in turn release GABA onto their targets in the deep cerebellar nuclei. But, as previously cited, *in vitro* application of NMDA to Purkinje cells might be paradoxically inhibitory [57]. Another complexity is that acute drug effects on cerebellar circuits fail to engage long-term mechanisms. Therefore chronic drug exposure, or chronic physiological effects such as loss of afferent drive, might have rather different consequences than acute exposure. For example, repeated electrical stimulation of climbing fibers in the presence of either exogenous glutamate or simultaneous parallel fiber stimulation, produces long-term depression of Purkinje cells [50]. Interestingly, in the present experiment, the agonist cocktail administered to exposed rats with nonsignificant tinnitus did not produce significant tinnitus until more than 300 hrs after the start of drug infusion (Fig. 8). But the drug-enhanced tinnitus then persisted for at least 30 days post drug, at which time testing was discontinued (Fig. 8, lower right panel). This suggests that cerebellar circuits in animals with minimal tinnitus were in a labile state, and that exposure to glutamatergic agonists permanently altered their function.

In the present study, the effects of glutamatergic antagonists and agonists were consistent with the hypothesis that glutamatergic circuits in the PFL contribute to chronic tinnitus. How likely was it that UBCs significantly contributed to that process? Granule cells constitute about one third of the cerebellar mass [53] and like UBCs, are glutamatergic, but unlike UBCs they synapse with the dendritic arbors of Purkinje cells, via parallel fibers and are not DCX positive. UBC DCX IR in the present experiment suggests a long-term plasticity of the UBC population in the adult brain in association with tinnitus. While granule cell-Purkinje cell interactive plasticity is well known, the majority of functional studies have characterized this plasticity in terms of rapid short-term effects [58]. Further experiments will be necessary to elucidate individual cerebellar components of chronic tinnitus.

Functional imaging in humans with chronic tinnitus and profound hearing loss has implicated the cerebellum as a structure significantly involved in generating the tinnitus percept [59]. However, a link between altered UBC function in either the cerebellum or the cochlear nucleus and tinnitus in humans is unknown. Although UBCs have been documented within the human cerebellum and cochlear nucleus, little is known about their relationship to pathological conditions. The results presented here suggest that the functional role of cerebellar UBCs in humans should be further investigated.

Finally the present findings may be considered in the larger context of tinnitus and hyperacusis. Hyperacusis refers to sound hypersensitivity. Clinically, hyperacusis can be measured using different methods, without complete agreement as to the optimum method. In animals, hyperacusis has been measured as an increase in the sensitivity to moderate-level sound pulses [60]. The prevalence of hyperacusis within the larger population of people with chronic tinnitus is unknown. Estimates range from 40–80% but no systematic survey has been conducted and therefore the true prevalence is unknown [61], [62], [63]. The psychophysical method used in the present experiments may reflect hypersensitivity to sound when discrimination functions are upshifted following acoustic trauma. Animals with this type of data profile were not included in the present report, and as such the results were interpreted in the context of tinnitus. It was also found that the glutamatergic agonists produced a discrimination function downshift, more consistent with a tinnitus interpretation than a hyperacusis interpretation. Nevertheless it is possible that following acoustic trauma a glutamatergic compensatory continuum emerges. Glutamatergic activation may produce hyperacusis, or a combination of tinnitus and hyperacusis, depending upon the level of activation.
